# Ice-Cream

**Published:** 1871-08

**Authors:** 


### ICE-CREAM.

A plate of ice-cream taken leisurely while seated at a table in pleasurable conversation, is a far safer quencher of thirst than a glass of ice-water, or any other ice-cold liquid; the ice-cream is, in addition, stimulating and nutritious, thus invigorating, cooling, and strengthening the system at the same time.

Ice-cream should not be taken immediately after a full meal, unless in the most leisure manner possible, — a plate full in the course of fifteen minutes, during lively conversation. If eaten rapidly it cools the stomach, prevents digestion, and causes acidity, unseemly belchings, if not actual chill, which in feeble persons endangers life.

